# Endothelial VEGFR-2 Activation Precedes Severe Mucosal Injury in TNBS-Induced Colitis

**DOI:** 10.3390/ijms27135810

**Published:** 2026-06-27

**Authors:** Sabrina Ceccariglia, Diego Sibilia, Alice Scattolini, Valentina Saccone, Ornella Parolini, Alessandro Armuzzi, Alfredo Papa, Antonio Gasbarrini, Fabrizio Pizzolante

**Affiliations:** 1 Department of Life Science and Public Health, Università Cattolica del Sacro Cuore, 00168 Rome, Italy; sabrina.ceccariglia@unicatt.it (S.C.); diego.sibilia1@unicatt.it (D.S.); alice.scattolini@unicatt.it (A.S.); valentina.saccone@unicatt.it (V.S.); ornella.parolini@unicatt.it (O.P.); 2 Fondazione Policlinico Universitario Agostino Gemelli IRCCS, 00168 Rome, Italy; 3 Fondazione IRCCS, Casa Sollievo della Sofferenza, San Giovanni Rotondo, 71013 Foggia, Italy; 4 IBD Center IRCSS, Humanitas Research Hospital, Rozzano, 20089 Milan, Italy; alessadro.armuzzi@hunimed.eu; 5 CEMAD (Digestive Disease Center), Department of Medical and Surgical Sciences, Fondazione Policlinico Universitario Agostino Gemelli IRCCS, 00168 Rome, Italy; alfredo.papa@unicatt.it (A.P.); antonio.gasbarrini@unicatt.it (A.G.); 6 Department of Translational Medicine and Surgery, Università Cattolica del Sacro Cuore, 00168 Rome, Italy

**Keywords:** TNBS colitis, VEGF-A expression, VEGFR-2 activation, endothelial response, inflammatory bowel disease

## Abstract

Endothelial VEGFR-2 plays a central role in vascular remodeling during intestinal inflammation, yet its activation during the early stages of colitis remains poorly characterized. Because Akt is a major downstream effector of VEGFR-2 signaling and a key mediator of endothelial responses, we investigated whether VEGFR-2 phosphorylation and Akt activation occur during the early phase of TNBS-induced colitis before the development of extensive mucosal injury. Acute colitis was induced in adult female Wistar rats by intracolonic administration of TNBS. Colonic tissues were collected on days 2, 4, and 6 after induction. Histological analyses and macrophage (CD68^+^ cells) infiltration were performed to characterize disease progression. VEGFR-2 expression and phosphorylation at Tyr1175 were evaluated on day 4 by Western blot, immunoprecipitation, and immunofluorescence. Akt activation was also assessed. TNBS-induced colitis is characterized by histological injury and increased CD68^+^ macrophage infiltration on day 4, with severe tissue damage observed on day 6. On day 4, colitis is associated with increased endothelial VEGFR-2 expression, enhanced VEGFR-2 phosphorylation at Tyr1175, and Akt activation. Early TNBS-induced colitis is associated with endothelial VEGFR-2 phosphorylation and Akt activation before the onset of extensive mucosal destruction on day 6. These findings support activation of the VEGFR-2/Akt signaling axis as an early vascular response during intestinal inflammation and suggest its potential contribution to disease progression.

## 1. Introduction

Inflammatory bowel diseases (IBD), primarily Crohn’s disease and ulcerative colitis, are chronic immune-mediated disorders of the gastrointestinal tract with increasing global incidence. Their pathogenesis arises from complex interactions among epithelial cells, resident and circulating immune cells, the gut microbiota, and the intestinal microvasculature, collectively leading to tissue injury and impaired mucosal homeostasis [1,2].

Pathological angiogenesis is increasingly recognized as a key contributor to IBD pathophysiology [3]. Although Crohn’s disease and ulcerative colitis exhibit distinct clinical and histopathological features, dysregulated angiogenic pathways are a shared hallmark across IBD subtypes. Expansion of the vascular network facilitates leukocyte recruitment and enhances the delivery of inflammatory mediators, while increasing microvascular and epithelial permeability, thereby sustaining mucosal inflammation and contributing to disease chronicity [1].

Among angiogenic mediators, vascular endothelial growth factor-A (VEGF-A) plays a central role in coordinating vascular responses [4,5]. VEGF-A exerts its effects primarily through vascular endothelial growth factor receptor-2 (VEGFR-2), a key regulator of endothelial function and inflammatory vascular remodeling [6]. Upon ligand binding, VEGFR-2 undergoes autophosphorylation and activates multiple downstream signaling cascades [7]. Among these, the Akt pathway represents one of the best-characterized and functionally critical downstream effectors of VEGFR-2 signaling, mediating key endothelial responses such as cell survival, vascular permeability, migration, and angiogenic remodeling [8,9,10,11]. Importantly, Akt activation is widely used as a canonical readout of VEGF-driven endothelial signaling and has been implicated in pathological angiogenesis and endothelial dysfunction in inflammatory bowel disease (IBD). Therefore, Akt signaling was selected as a downstream marker to evaluate the functional consequences of early VEGFR-2 activation in our experimental setting.

Elevated VEGF-A levels have been reported in patients with ulcerative colitis and in experimental models, supporting its involvement in intestinal inflammation [12,13]. A recent systematic review and meta-analysis confirmed that circulating VEGF levels are significantly increased in IBD patients compared with controls, with higher levels observed in ulcerative colitis than in Crohn’s disease [14].

Despite this evidence, whether vascular activation represents an early inflammation driver or a secondary response remains unresolved. Emerging data suggest a potential causal role for VEGF signaling in IBD pathogenesis [15], and indicate that endothelial activation may precede overt tissue injury [16]. However, although VEGF-A overexpression in colitis is well documented, early VEGFR-2 activation and its functional consequences remain poorly characterized. This gap is particularly relevant during the initial phases of inflammation, before the onset of overt histological damage, when endothelial signaling may contribute to disease initiation and amplification. A better understanding of these early vascular events may identify novel therapeutic targets aimed at modulating angiogenesis to limit intestinal inflammation.

In this study, we investigated VEGFR-2 expression and phosphorylation in intestinal endothelial cells in a Trinitrobenzene sulfonic acid (TNBS)-induced rat model of acute colitis. This model recapitulates early inflammatory and vascular alterations, allowing the investigation of endothelial responses prior to extensive tissue damage [17,18,19,20]. Specifically, we assessed whether VEGFR-2 activation occurs early during disease progression, before the development of overt histological injury typically associated with chronic colitis [21,22], with a particular focus on downstream Akt activation.

## 2. Results

### 2.1. Characterization of Colon Mucosal Damage in TNBS-Induced Colitis

#### 2.1.1. Macroscopic and Histological Evaluation

TNBS-induced colitis is a well-established experimental model of intestinal inflammation and is widely used to investigate pathogenic mechanisms of experimental colitis [23].

**Figure 1:**
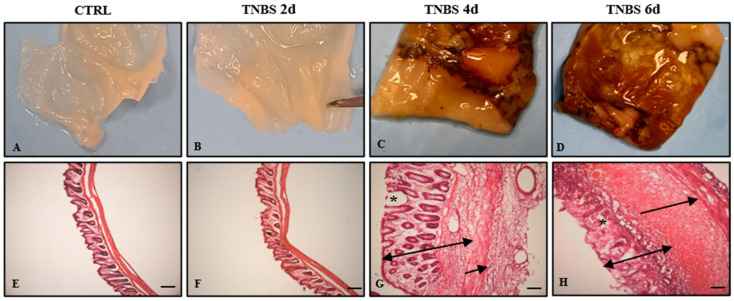
Macroscopic and histological features of colonic mucosa in TNBS-treated rats. Representative macroscopic images (**A**–**D**) and H&E-stained sections (**E**–**H**) of colon from control (**A**,**E**) and TNBS-treated rats (**B**–**D**,**F**–**H**). After 2 days of TNBS treatment, no consistent macroscopic or histological alterations are observed (**B**,**F**). On day 4, increased colonic wall thickness and focal mucosal necrosis are evident macroscopically (**C**), while histological analysis reveals initial epithelial damage (asterisk), mucosal (double-headed arrows) and submucosal edema (arrow), and inflammatory cell infiltration (**G**). On day 6, severe colonic injury is observed, characterized by marked wall thickening and extensive mucosal necrosis (**D**), together with severe epithelial damage (asterisk), pronounced mucosal (double-headed arrows) and submucosal edema (arrow), loss of normal tissue architecture, and extensive inflammatory cell infiltration (**H**). Scale bars: 50 μm. CTRL: control; TNBS: trinitrobenzene sulfonic acid; d: days. For interpretation of the references to color in this figure legend, the reader is referred to the Web version of this article.

Macroscopic examination of colonic tissue from control (Figure 1A) and treated animals at day 2 (Figure 1B) revealed preserved morphology. Upon longitudinal opening, the mucosal surface was regular and translucent, with no visible erosion, necrosis, or hemorrhagic areas. In contrast, colonic tissue from TNBS-treated animals at day 4 (Figure 1C) showed morphological alterations, including increased wall thickness, irregular mucosa, necrosis regions alternating with preserved areas and focal hemorrhagic regions, consistent with a patchy distribution of inflammation. Colonic tissue from TNBS-treated animals on day 6 (Figure 1D) exhibited severe injury, characterized by extensive mucosal necrosis, marked thickening of the colonic wall, and profound disruption of normal colonic architecture.

Histological evaluation of hematoxylin and eosin (H&E)-stained colon sections revealed preserved mucosal architecture in control animals (Figure 1E) and in TNBS-treated animals at day 2 (Figure 1F). In contrast, TNBS-treated animals at day 4 exhibited morphological alterations, including partial epithelial disruption (asterisk), mucosal (double-headed arrows) and submucosal edema (arrow), and inflammatory cell infiltration (Figure 1G). On day 6, TNBS-treated animals showed markedly aggravated histological damage (Figure 1H), characterized by severe epithelial destruction (asterisk), loss of normal crypt architecture, pronounced submucosal edema (arrow), and extensive inflammatory cell infiltration. These findings are consistent with the progressive development of acute TNBS-induced colitis. Histopathological alterations were further assessed using the semiquantitative scoring system described in the Materials and Methods section (Table 1). Consistent with the morphological observations, histological scores increased progressively from day 2 to day 6, confirming the time-dependent worsening of TNBS-colonic injury.

As an additional indicator of disease severity, TNBS-treated animals exhibited a progressive reduction in body weight relative to baseline values, reaching approximately 0–1%, 5–6%, and 8–9% weight loss at days 2, 4, and 6 after colitis induction, respectively.

#### 2.1.2. Macrophage Infiltration

**Figure 2:**
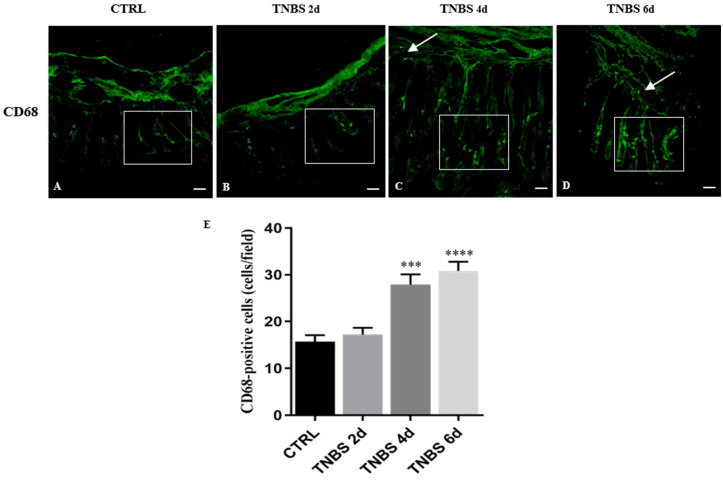
Macrophage infiltration in the colon of TNBS-treated rats. Immunofluorescence staining for CD68 (green; **A**–**D**) shows increased macrophage infiltration in the colonic mucosa of treated rats at 4 and 6 d (**C**,**D**) compared with controls (**A**) and 2 d (**B**). Squares and arrows highlight clusters of CD68-positive cells. Scale bars: 25 μm. Quantification of CD68-positive cells (**E**) confirms a significant increase in macrophage infiltration after TNBS administration at 4 and 6 d. Data are presented as mean ± SEM. *** *p* ≤ 0.001 and **** *p* ≤ 0.0001 vs. CTRL. CTRL: control, TNBS: trinitrobenzene sulfonic acid, d: days. For interpretation of the references to color in this figure legend, the reader is referred to the Web version of this article.

To further characterize the inflammatory response, macrophage infiltration was assessed by immunofluorescence staining for CD68 in colonic tissue from control and TNBS-treated animals (Figure 2A–D).

In control (Figure 2A) and treated colons at day 2 (Figure 2B), CD68-positive cells were few and localized within the mucosa (boxed area), showing a scattered distribution consistent with resident macrophages under physiological conditions. The fluorescence signal was weak and confined to discrete cellular profiles, with preserved mucosal architecture. In contrast, treated animals at day 4 (Figure 2C) showed a pronounced increase in CD68 immunoreactivity. Numerous CD68-positive cells were observed throughout the mucosa (boxed area) and extended into the submucosa (arrow), indicating enhanced macrophage infiltration. On day 6 (Figure 2D) CD68 immunoreactivity was further enhanced with a greater density of CD68-positive cells distributed throughout the damaged colonic tissue. The fluorescence signal was intense and widespread, with clustering of positive cells particularly in areas corresponding to mucosal damage.

Quantitative analysis confirmed a significant increase in CD68-positive cells in treated animals at day 4 and 6 compared with control and 2 d (respectively *** *p* ≤ 0.001 and **** *p* ≤ 0.0001) (Figure 2E).

Overall, these findings indicate robust inflammatory cell recruitment and macrophage infiltration in TNBS-induced colitis on day 4 and 6. Although macrophage activation was not directly evaluated, previous studies using the same TNBS-induced colitis model reported increased expression of the pro-inflammatory cytokines TNF-α and IL-1β, which are characteristic mediators produced by activated M1 macrophages [17,24,25]. These observations support the presence of a pro-inflammatory environment associated with macrophage cells under these experimental conditions.

### 2.2. Analysis of Angiogenic Mediators in the Colon After TNBS Treatment

#### 2.2.1. Expression of RECA-1, VEGF-A and VEGFR-2 Proteins

To investigate vascular changes and VEGF-related mediators in TNBS-induced colitis (4 days), we evaluated the expression of rat endothelial cell antigen-1 (RECA-1), VEGF-A and VEGFR-2 by Western blot analyses of colonic tissue homogenates.

**Figure 3:**
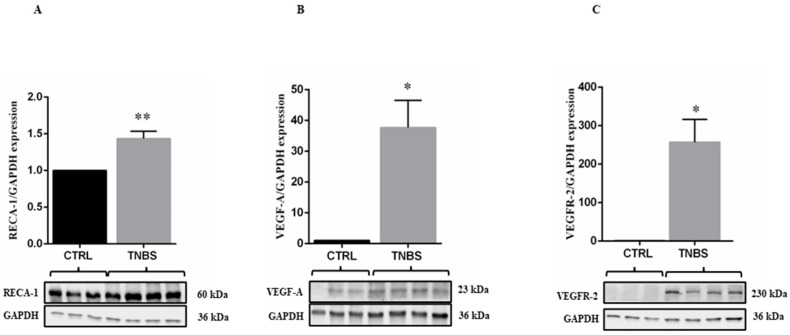
Expression of RECA-1, VEGF-A, and VEGFR-2 Levels in the Colon of TNBS-Treated Rats. Representative Western blot analysis and densitometric quantification of RECA-1 (**A**), VEGF-A (**B**), and VEGFR-2 (**C**) in colonic tissue showing increased protein expressions in treated rats compared with controls. Protein levels were normalized to GAPDH and expressed as fold change relative to the control group, which was set to 1. Data were expressed as fold change relative to the control group, which is set to 1, and presented as mean ± SEM. * *p* ≤ 0.05 and ** *p* ≤ 0.01 vs. CTRL. CTRL: control, TNBS: trinitrobenzene sulfonic acid.

RECA-1 was significantly increased in treated animals compared with controls (** *p* ≤ 0.01) (Figure 3A), consistent with increased vascularization.

VEGF-A protein levels were significantly elevated in treated animals relative to controls (* *p* ≤ 0.05) (Figure 3B).

Finally, VEGFR-2 was also significantly upregulated in treated animals compared to controls (* *p* ≤ 0.05) (Figure 3C), reflecting a pro-angiogenic response.

Collectively, these findings indicate that TNBS-induced colitis is associated with upregulation of the VEGF-A/VEGFR-2 axis and increased vascular presence in the colon.

#### 2.2.2. VEGFR-2 Localization in Vascular Endothelial Cells

To assess VEGFR-2 distribution and cellular localization, double immunofluorescence staining for VEGFR-2 and RECA-1 was performed on colonic sections.

**Figure 4:**
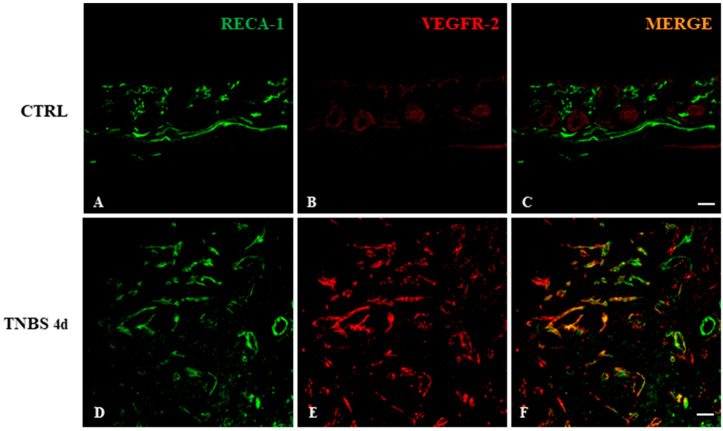
Localization of RECA-1 and VEGFR-2 in the colon of TNBS-treated rats. Double-labeled immunofluorescence micrographs show enhanced RECA-1 (green; **D**) and VEGFR-2 (red; **E**) immunoreactivity in the colonic mucosa of treated rats (**D**–**F**) compared with control sections (**A**–**C**), with prominent colocalization in merged image of treated rats (**F**). Scale bars: 25 μm. Abbreviations: CTRL: control, TNBS: trinitrobenzene sulfonic acid, d: days. For interpretation of the references to color in this figure legend, the reader is referred to the Web version of this article.

In treated animals (4 days), VEGFR-2 immunoreactivity was prominently detected in mucosal vascular structures showing clear and marked colocalization with RECA-1–positive endothelial cells (Figure 4F). Consistent with Western blot findings, RECA-1 labeling was visibly increased (Figure 4D), and VEGFR-2 staining was markedly diffused (Figure E). Control colon sections exhibited limited RECA-1 immunoreactivity (Figure 4A), whereas VEGFR-2 staining was minimal or undetectable (Figure 4B).

#### 2.2.3. Molecular VEGFR-2 and Akt Activation in the Colon Mucosa After TNBS Colitis

We next investigated whether VEGFR-2 upregulation in treated animals was associated with his activation. VEGFR-2 undergoes autophosphorylation upon ligand binding, a prerequisite for downstream signaling [26].

**Figure 5:**
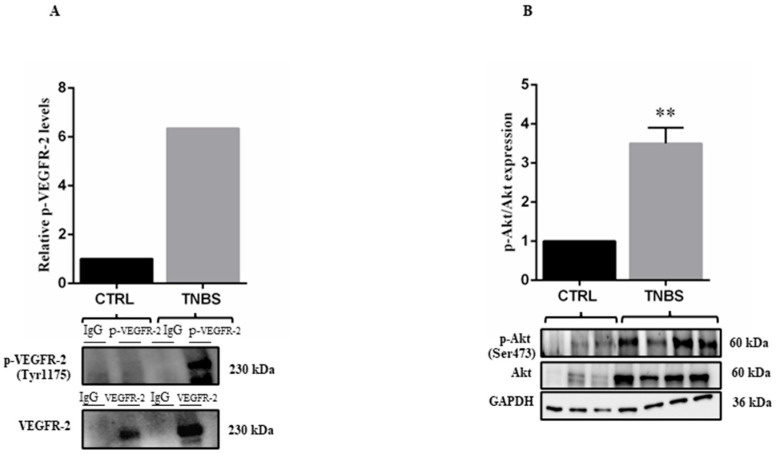
VEGFR-2 phosphorylation and Akt activation in the colon of TNBS-treated rats. Representative immunoprecipitation of VEGFR-2 followed by Western blot analysis and densitometric quantification of p-VEGFR-2 relative to total VEGFR-2 (**A**) in colonic tissue from control and treated rats, showing increased VEGFR-2 phosphorylation in treated rats compared with controls. Representative Western blot analysis and densitometric quantification of p-Akt relative to total Akt (**B**) demonstrate increased Akt activation in treated rats compared with controls. p-VEGFR-2 and p-Akt/Akt ratio levels were normalized respectively to the total VEGFR-2 and GAPDH. Data were expressed as fold change relative to the control group, which is set to 1, and presented as mean ± SEM. ** *p* ≤ 0.01 vs. CTRL. CTRL: control, TNBS: trinitrobenzene sulfonic acid, IgG: immunoglobulin G.

VEGFR-2 autophosphorylation was assessed by immunoprecipitation of VEGFR-2 experiment followed by Western blot analysis for p-VEGFR-2 using pooled colonic homogenates (CTRL, *n* = 3; TNBS, *n* = 4) to ensure reliable detection of low-abundance VEGFR-2 in control samples as demonstrated by previous Western blot findings. TNBS treatment (4 days) resulted in a fivefold increase in VEGFR-2 phosphorylation compared with controls (Figure 5A).

Successively, to investigate Akt activation, total Akt and p-Akt were measured in individual colonic homogenates [27,28]. Total Akt expression was increased in treated animals compared with controls, and the p-Akt/Akt ratio, reflecting kinase activation, was significantly elevated (** *p* ≤ 0.01) (Figure 5B).

These findings indicate that TNBS-induced colitis is associated with functional engagement of VEGFR-2, as evidenced by increased receptor phosphorylation and downstream Akt activation.

## 3. Discussion

Early intestinal inflammation in TNBS-induced colitis is associated with activation of the endothelial VEGF-A/VEGFR-2 signaling axis. Beyond the observed increase in VEGF-A and VEGFR-2 expression, the marked phosphorylation of VEGFR-2 at Tyr1175 together with enhanced Akt activation indicates that this pathway is functionally engaged during the early stages of disease development. These findings suggest that angiogenic signaling is not merely upregulated at the expression level but is actively transduced within the intestinal vasculature, supporting the concept of an early endothelial response accompanying the development of mucosal inflammation.

This early signaling activation occurs within a pro-angiogenic inflammatory milieu characterized by increased VEGF-A expression. VEGF-A upregulation, widely reported in both experimental colitis and human IBD [13,29,30], may provide the upstream stimulus for VEGFR-2 activation in the inflamed mucosa. Our findings extend this concept by demonstrating that ligand availability is coupled with receptor phosphorylation and activation, thereby defining a functional angiogenic switch rather than a static increase in expression.

TNBS colitis is a well-established chemically induced model that reproduces key histopathological features of human IBD, including epithelial damage, leukocyte infiltration, and transmural inflammation [23,31]. Although often considered Crohn’s disease–like, it provides a controlled framework to investigate early inflammatory events before advanced tissue destruction.

Histologically, the four-day time point represents a transitional phase characterized by epithelial injury, mucosal inflammation, and submucosal edema, while overall tissue architecture remains preserved. This stage was selected based on macroscopic and histological observations showing inconsistent changes at earlier time points (day 2) and precedes the extensive damage observed at later stages (day 6). It therefore provides a suitable temporal window to capture early signaling events without confounding effects of advanced tissue destruction.

Within this context, in TNBS-treated colonic tissue, the progressive accumulation of CD68^+^ macrophages is consistent with the development of an active inflammatory microenvironment. Macrophages have been reported to produce VEGF-A during intestinal inflammation [32] However, the present study was not designed to identify the cellular source of VEGF-A expression, and therefore no conclusions can be drawn regarding the relative contribution of macrophages or other cell populations to the increased VEGF-A levels observed in TNBS colitis.

At the tissue level, increased RECA-1 and VEGF-A expressions were associated with upregulation of VEGFR-2 supporting early endothelial activation and expansion of the vascular compartment. Importantly, VEGFR-2 localization within endothelial structures indicates the notion that signaling activation occurs directly within the vascular niche rather than being restricted to paracrine or systemic effects. In this context, endothelial cells integrate inflammatory cues and coordinate vascular and immune responses [33].

Angiogenesis in intestinal inflammation should not be viewed solely as a late structural remodeling process but also as an early functional response integrating vascular and immune signaling [3,34,35].

In this study, VEGFR-2 activation is associated with early vascular remodeling, suggesting that angiogenic signaling is engaged at an early stage and may be relevant to processes involved in leukocyte trafficking and tissue adaptation prior to the development of extensive histological damage.

Mechanistically, phosphorylation of VEGFR-2 at Tyr1175 is particularly relevant as this residue serves as a major docking site for downstream signaling complexes involved in Akt pathway activation [36,37,38]. Consistently, increased Akt phosphorylation supports activation of endothelial survival and migration programs, processes essential for early vascular adaptation during inflammation.

Our findings demonstrate an association between intestinal inflammation, VEGFR-2 phosphorylation, and Akt activation within the endothelial compartment, supporting the presence of an early angiogenic signaling response. Together, these data are consistent with the presence of an “angiogenic switch”, whereby endothelial cells acquire an activated phenotype accompanied with inflammatory and vascular responses.

This study has limitations. The relatively small sample size may limit statistical power and generalizability of the findings. However, the observed effects were consistent across animals within each group and across multiple independent readouts. Moreover, similar sample sizes have been widely used in previous TNBS-induced colitis studies reporting robust and reproducible biological effects [39,40,41], supporting the methodological validity of this experimental approach. The acute TNBS model does not fully recapitulate the complexity of chronic human IBD. Moreover, the correlative nature of the data precludes definitive causal inference regarding VEGFR-2 signaling. Additional downstream pathways, such as PLCγ and ERK signaling, were not investigated and may also contribute to the observed phenotype.

In conclusion, we identify early activation of endothelial VEGF-A/VEGFR-2 signaling axis in TNBS-induced colitis, characterized by receptor phosphorylation and downstream Akt activation. These findings indicate that endothelial VEGF-A/VEGFR-2 signaling is engaged during the early phase of intestinal inflammation and is associated with activation of downstream signaling pathways. Collectively, our results support the concept that the intestinal endothelium participates in early inflammatory responses rather than functioning solely as a passive target of inflammation.

Further studies employing pharmacological or genetic inhibition of VEGFR-2, together with validation in human IBD tissues are required to determine whether modulation of the VEGF-A/VEGFR-2 axis may influence disease progression and represent a therapeutically relevant target in IBD.

## 4. Materials and Methods

### 4.1. Animal Model

#### 4.1.1. Animals

Adult female *Wistar* rats (200–250 g, 2 months old) were obtained from the institutional animal facility of the Catholic University of Rome (Italy). Rats were individually housed in an air-conditioned room at 25 °C, with free access to food and water and maintained under a standardized light/dark cycle.

#### 4.1.2. Induction of Experimental Colitis

Experimental colitis was induced by once and slow intracolonic administration of TNBS (2,4,6-trinitrobenzenesulfonic acid; Sigma-Aldrich, St. Louis, MO, USA) at a dose of 60 mg/kg dissolved in 0.3 mL of 50% ethanol. TNBS was delivered through a silicone catheter inserted 6 cm proximally from the anus, as previously described [17]. After administration, rats were maintained in a head-down position for approximately 60 s to prevent reflux. Control rats received 0.3 mL of saline. Animals were weighed daily, as weight loss is correlated with increases in severity of colitis [39].

#### 4.1.3. Experimental Design

Rats were euthanized at 2, 4, and 6 days following TNBS administration. Control rats (*n* = 3) and TNBS groups (*n* = 4 per time point) were anesthetized by intraperitoneal injection of ketamine (87 mg/kg; Ketavet 100, Merck Sharp & Dohme, Rahway, NJ, USA) and xylazine (12.5 mg/kg; Rompum, Bayer, Leverkusen, Germany) and then euthanized by decapitation. The smaller sample size in the control group reflects the reduced biological variability typically observed in untreated animals, whereas a larger number of TNBS-treated animals was included to account for the expected variability in disease severity across time points. During autopsy, a 5-cm segment of distal colon was collected for morphological and molecular analyses. Although sample sizes were limited, they are consistent with exploratory mechanistic studies and were sufficient to detect early molecular changes associated with acute inflammation.

The number of animals per group was minimized in accordance with the principles of the 3Rs (Replacement, Reduction, and Refinement). Reduction was achieved by optimizing the experimental design and performing multiple complementary analyses on the same tissue samples, thereby limiting the total number of animals required. Refinement strategies included the use of standardized anesthesia protocols and careful monitoring to minimize animal distress.

### 4.2. Macroscopic and Histopathological Evaluation of Colonic Damage

Colon tissues from control and TNBS-treated animals (days 2, 4, and 6 after colitis induction) were collected, rinsed with saline, and photographed for macroscopic assessment. For histological analysis, samples were fixed overnight in 10% buffered formalin at 4 °C, cryoprotected in 30% sucrose, embedded in OCT compound, and sectioned at 5 μm thickness using a Leica CM1850 cryostat (Leica Microsystems, GmbH, Wetzlar, Germany) at −20 °C. Sections were mounted onto glass slides, air-dried, and stained with hematoxylin and eosin (H&E) according to standard protocols. Images were acquired using a Zeiss Axiophot microscope (Carl Zeiss AG, Oberkochen, Germany). Histopathological evaluation was performed by investigator blinded to the experimental groups. Colonic injury was assessed qualitatively and by a semiquantitative histological scoring system based on epithelial damage, inflammatory cell infiltration, edema, crypt destruction, and lesion extent, as previously described by Dieleman et al. [42], with minor modifications. Each parameter was scored from 0 to 3 according to the severity of the observed histopathological alterations, as detailed in Table 1. The total histological score was calculated as the sum of the individual parameter scores, yielding a maximum score of 15, with higher scores indicating greater tissue damage.

### 4.3. Western Blot Analysis of RECA-1, VEGF-A, VEGFR-2 and p-Akt/Akt Expression

Western blot analyses were performed in control rats and TNBS-treated animals on day 4. Colons were collected and stored at −80 °C. Tissue samples were homogenized in ice-cold lysis buffer (50 mM Tris-HCl, pH 7.4, 150 mM NaCl, 1% Triton X-100, 0.1% SDS, 0.5% sodium deoxycholate) supplemented with protease (Sigma-Aldrich, Cat. #P8849) and phosphatase (Sigma-Aldrich, St. Louis, MO, USA, Cat. #P0044) inhibitor cocktails. After lysates were incubated on ice for 30 min and centrifuged at 14,000× *g* for 30 min at 4 °C. Protein concentration was determined using a micro-BCA protein assay (Thermo Fisher Scientific, Waltham, MA, USA, Cat. #23227). Equal amounts of proteins (30 μg/lane) were separated by SDS-PAGE (5% or 10%) (Bio-Rad Laboratories, Hercules, CA, USA, Cat. #4561024 and #4561034) and transferred to PVDF membranes (Bio-Rad Laboratories, Hercules, CA, USA, Cat. #1704156). Membranes were blocked for 1 h at room temperature with 5% non-fat dry milk or 5% BSA in TBS-T depending on the target and, incubated overnight at 4 °C with primary antibodies against RECA-1 (Invitrogen, Cat. #MA1-81510; 1:700), VEGF-A (Abcam, Cambridge, UK, Cat. #ab46154; 1:1000), VEGFR-2 (Cell Signaling Technology, Cat. #55B11; 1:1000), total Akt (Cell Signaling Technology, Inc., Danvers, MA, USA, Cat. #11E7; 1:1000), phospho-Akt (Ser473) (Cell Signaling Technology, Inc., Danvers, MA, USA, Cat. #193H12; 1:1000), and GAPDH (Invitrogen, Waltham, MA, USA, Cat. #MA5-15738; 1:2000). After washing, sections were incubated for 1 h at room temperature with secondary HRP-conjugated antibodies (Invitrogen, Waltham, MA, USA, Cat. #A16023 and Cat. #A24500; 1:1000-1:2000). Signals were detected using enhanced chemiluminescence (Bio-Rad Laboratories, Hercules, CA, USA, Cat. #1705060) and visualized with a ChemiDoc Imaging System (Bio-Rad Laboratories, Hercules, CA, USA).

Densitometric analysis was performed using ImageJ (NIH, version 1.53 k). Protein levels were normalized to GAPDH and expressed as fold change relative to control group, which was set to 1.

All Western blot analyses were performed in at least three independent experiments.

### 4.4. Immunofluorescence Localization of RECA-1, VEGFR-2 and CD68 Proteins

Control and TNBS-treated rat colons on day 4 for RECA-1 and VEGFR-2 immunostaining and at days 2, 4, and 6 for CD68 immunofluorescence were collected, fixed overnight in 4% paraformaldehyde at 4 °C and rinsed in PBS, pH 7.4. Transverse sections (25 μm) were obtained using the cryostat. For RECA-1 and VEGFR-2 colocalization, and macrophage detection, sections were blocked with 5% normal donkey serum (Jackson ImmunoResearch Laboratories, West Grove, PA, USA, Cat. #017-000-001) for 1 h at room temperature and incubated overnight at 4 °C respectively with primary antibodies against RECA-1 (Thermo Fisher Scientific, Waltham, MA, USA, Cat. #MA1-81510; 1:200), VEGFR-2 (Cell Signaling Technology, Inc., Danvers, MA, USA, Cat. #2479; 1:200) and CD68 (Abcam, Cambridge, UK, Cat. #ab955; 1:300). After washing, sections were incubated for 1 h at room temperature with donkey anti-mouse Alexa Fluor 488 (Jackson ImmunoResearch Laboratories, West Grove, PA, USA, Cat. #715-545-150; 1:250) and donkey anti-rabbit Cy3 (Jackson ImmunoResearch Laboratories, West Grove, PA, USA, Cat. #711-165-152; 1:400) secondary antibodies. Finally, all sections were mounted using Fluoromount medium (Sigma-Aldrich, St. Louis, MO, USA, Cat. #F4680).

Images were acquired using a Nikon Eclipse Ti2 confocal microscope (Nikon Corporation, Tokyo, Japan) under identical settings across groups.

Negative controls were obtained by omitting primary antibodies. Five non-consecutive sections per animal were analyzed.

#### Quantification of CD68-Positive Macrophages

CD68-positive cells were quantified in colonic sections of control and TNBS-treated rats at days 2, 4 and 6. Multiple non-consecutive sections per animal were analyzed. Cells were counted in eight randomly selected fields per section (0.021 mm^2^ each), using a Zeiss Axiophot microscope equipped with AxioVision Rel 4.5 (Carl Zeiss MicroImaging GmbH, Jena, Germany). CD68-positive cells were identified based on cytoplasmic staining and morphological characteristics consistent with macrophages in line with established immunohistochemical approaches [43].

Data were expressed as the number of positive cells per field and average per animal. Analysis was performed by a blind investigator; automated quantification was not used.

### 4.5. pVEGFR-2 Level Expression

Colon tissues from control rats and TNBS-treated animals on day 4 were homogenized in ice-cold lysis buffer (10 mM Tris-HCl, pH 7.5, 150 mM NaCl, 5 mM EDTA, 0.5% NP-40) supplemented with protease (Complete^TM^, EDTA-free, Roche, Mannheim, Germany, Cat. #11836153001) and phosphatase (PhosSTOP^TM^, Roche, Mannheim, Germany, Cat. #04906837001) inhibitor cocktails. Lysates were incubated on ice for 30 min, sonicated (30 s ON/30 s OFF for 10 min, high intensity; BIORUPTOR, Denville, NJ, USA) and centrifuged at 14,000× *g* for 20 min at 4 °C. Protein concentration was determined using a micro-BCA protein assay (Thermo Fisher Scientific, Waltham, MA, USA, Cat. #23235).

Due to the low expression of VEGFR-2 in control samples, lysates were pooled within each group to obtain sufficient protein for immunoprecipitation. This approach limited statistical power and precluded assessment of inter-individual variability and is therefore acknowledged as a methodological limitation.

Ten percent of each lysate was kept as input control. For immunoprecipitation, 1 mg of protein was incubated overnight at 4 °C with anti-VEGFR-2 antibody (Cell Signaling Technology, Danvers, MA, USA, Cat. #2479; 1:100) or control IgG (Santa Cruz Biotechnology, Dallas, TX, USA, Cat. #sc-66931; 1:100). Immune complexes were captured performed using 100 μL of Protein G Dynabeads (Thermo Fisher Scientific, Waltham, MA, USA, Cat. #10004D) and washed extensively [44]. Immunoprecipitated proteins were resuspended in 2× Laemmli buffer and boiled at 95 °C for 5 min followed by the addition of 4× Laemmli sample buffer (Bio-Rad, Laboratories, Hercules, CA, USA, Cat. #1610747). Samples were resolved on a 6% SDS–PAGE gel and transferred to PVDF membranes (Bio-Rad, Laboratories, Hercules, CA, USA, Trans-Blot^®^ Turbo™ Mini PVDF Transfer Packs, Cat. #170415). Membranes were blocked with 5% BSA in TBS-T and incubated overnight at 4 °C with primary antibodies against VEGFR-2 (Cell Signaling Technology, Danvers, MA, USA, Cat. #2479; 1:1000) and p-VEGFR-2 (Tyr1175) (Cell Signaling Technology, Danvers, MA, USA, Cat. #2478; 1:1000). Membranes then were incubated with secondary HRP-conjugated antibody for 1 h at room temperature.

Signals were detected using the enhanced chemiluminescence (Thermo Fisher Scientific, Waltham, MA, USA, Pierce™ ECL Western Blotting Substrate, Cat. # 32106) and visualized with a ChemiDoc Imaging System (Bio-Rad, Laboratories, Hercules, CA, USA).

Densitometric analysis was performed using ImageJ (NIH, version 1.53 k). The levels of p-VEGFR-2 were normalized to total VEGFR-2 and expressed as fold change relative to the control group, which was set to 1.

### 4.6. Statistical Analysis

Statistical analyses were performed using GraphPad Prism version 10.0 (GraphPad Software, Boston, MA, USA). Data are expressed as mean ± SEM. Histological scores are reported as mean ± SEM and were used for descriptive purposes only. The comparisons between two groups were performed using an unpaired Student’s *t*-test, whereas differences among multiple groups were assessed using one-way analysis of variance (ANOVA) followed by Dunnett’s multiple-comparison post hoc test. Statistical significance was defined as *p* ≤ 0.05 (*), *p* ≤ 0.01 (**), *p* ≤ 0.001 (***), and *p ≤* 0.0001 (****).

## Figures and Tables

**Table 1:** Histopathological score system in TNBS-induced colitis. Colonic injury is assessed using a semiquantitative scoring system. Data are expressed as mean ± SEM. Higher scores indicate greater histopathological damage. CTRL: control, TNBS: trinitrobenzene sulfonic acid, d: days.

Group	Epithelial Damage	Inflammatory Infiltration	Edema	Crypt Destruction	Lesion Extent	Total Score
**CTRL**	0.0 ± 0.0	0.0 ± 0.0	0.0 ± 0.0	0.0 ± 0.0	0.0 ± 0.0	0.0 ± 0.0
**TNBS 2d**	0.5 ± 0.3	0.0 ± 0.0	0.0 ± 0.0	0.0 ± 0.0	0.0 ± 0.0	0.5 ± 0.3
**TNBS 4d**	1.25 ± 0.25	1.75 ± 0.25	1.25 ± 0.25	0.0 ± 0.0	0.75 ± 0.25	5.0 ± 1.0
**TNBS 6d**	2.75 ± 0.25	2.75 ± 0.25	2.25 ± 0.25	3.0 ± 0.0	3.0 ± 0.0	13.75 ± 0.75

## Data Availability

All data generated or analyses during this study are included in this article.

## Abbreviations

H&E	Hematoxylin and eosin
IBD	Inflammatory bowel diseases
RECA-1	Antigen cell endothelial rat-1
TNBS	Trinitrobenzene sulfonic acid
VEGF-A	Vascular endothelial growth factor-A
VEGFR-2	Vascular endothelial growth factor receptor-2
